# “Youth are experts in what they need”: experiences and best practice in co-designing and implementing Fast-PrEP, a novel PrEP service for adolescents and youth in Cape Town, South Africa

**DOI:** 10.3389/fpubh.2024.1459418

**Published:** 2024-12-05

**Authors:** Fiona Bennin, Lauren Fynn, Pamela Fuzile, Ntando Yola, Robin Julies, Siyaxolisa Sindelo, Ndumiso Madubela, Prisca Vundhla, Yolanda Mpanda, Mbali Jonas, Linda-Gail Bekker, Elzette Rousseau

**Affiliations:** Desmond Tutu Health Foundation, University of Cape Town, Cape Town, South Africa

**Keywords:** PrEP (pre-exposure prophylaxis), adolescents and young people, co-design, YRG, youth-friendly

## Abstract

**Introduction:**

Defining the prevention needs, motivations, and gender dynamics influencing adolescent and young people’s (AYP’s) healthcare access is a key component to successful PrEP (pre-exposure prophylaxis for HIV prevention) implementation. WHO encourages a strong people-cantered approach to healthcare delivery, and this is particularly emphasized for HIV services. Enhanced youth engagement is needed to ensure that interventions are tailored to the specific needs and preferences of youth populations.

**Description:**

Fast-PrEP is an implementation science project providing PrEP (oral, vaginal ring, and injectable) to adolescents and young people (15–29 years old) in Cape Town, South Africa. In 2020, during the planning phase of this project, a Youth Reference Group (YRG) was established to guide, co-create, monitor, and evaluate the implementation of PrEP delivery. From March to October 2023, we conducted four focus group discussions with thirty YRG members, and seven interviews with project implementers (including clinical and research staff). This study aimed to explore real-time experiences of young people and staff members and provide recommendations for best practices when setting up and engaging with YRGs.

**Findings:**

Overall, young people described their experience of being involved in the YRG as empowering. Young people felt that their voices and needs were valued when seeing their input put into action or witnessing their designs in demand creation campaigns. Young people felt that being consulted was not for tokenistic reasons, but their contribution was valuable and considered young people’s sexual health needs. Best practices included involving the YRG in every aspect of the project design and implementation, being flexible around young people’s schedules, and having engagements in spaces that are easily accessible and safe for key populations. Staff recommended upskilling the youth members in knowledge around HIV and certain ‘soft skills’ such as building self-confidence and communication skills. It was also recommended that all research and clinical staff need ongoing training and sensitization on the importance and value of youth engagement.

**Conclusion:**

The involvement of young people in the co-creation of Fast-PrEP services in all phases of service provision, has been effective in assisting to provide youth appropriate PrEP services.

## 1 Introduction

HIV remains one of the most significant global public health challenges of our time. Of particular concern is the high prevalence of HIV among adolescents and young people (AYP) in Eastern and Southern Africa (1). To achieve an ambitious set of HIV prevention targets by 2030, strategies have been proposed to expand access to health services including the uptake and effective use of pre-exposure prophylaxis (PrEP) by AYP (2). While PrEP products for HIV prevention are highly effective and safe, a recent global systematic review showed that 47% of people in Eastern and Southern Africa discontinued PrEP within 6 months of initiation (3). Effective HIV prevention requires well-designed and well-targeted services tailored according to client characteristics and needs (4). To ensure PrEP reaches the right people, active collaboration and engagement with AYPs as end-users are crucial: involving them in the design, implementation, and evaluation of PrEP programmes to address their specific needs, preferences, and challenges related to HIV prevention.

The World Health Organisation (WHO) encourages universal healthcare access to all people when and where needed, beyond just the availability of healthcare resources (institutions, procedures, drug supply, and regulations), to include a robust people-centred approach (5). People-centred accessibility is an approach that prioritizes the needs and experiences of individuals seeking health services and includes approachability, acceptability, availability, affordability, and appropriateness of services (6). The same applies to PrEP services, where a shift toward differentiated PrEP service delivery has been observed in recent years, particularly during and after the COVID-19 pandemic (7). The four building blocks of differentiated PrEP service delivery include the location of services, choice of service providers, service frequency and what is included in the service package. This differentiated PrEP service delivery approach adapts to the needs of those using PrEP or those who could benefit from PrEP and can assist in providing more cost-effective and efficient use of healthcare resources. The WHO recommends that communities and end users need to be involved in the design, planning and delivery of these services (7). While there has been a recent inclusion of young people during engagements with community advisory boards (CABs), enhanced youth engagement is needed to ensure that interventions are tailored to the specific needs and preferences of youth populations (8, 9). Adolescents and young people have stated their desire to be involved in decisions regarding HIV, and engaging youth can have positive and large impacts on research, programming, demand generation for new HIV prevention products and ultimately an effective HIV response in this population (8, 10–12). More research is needed to identify successful strategies for including AYP in the development and evaluation of PrEP programmes.

The Fast-PrEP project was an implementation science project established to co-create, monitor, and evaluate PrEP provision (oral, dapivirine vaginal ring, and injectable cabotegravir) with a youth reference group. The Fast-PrEP study assessed the design and delivery of PrEP from differentiated service delivery platforms in a single health district in Cape Town, South Africa. In this manuscript, we share the experiences and best practices, as recommended by both study implementers and young people involved in the youth reference group, for co-designing, implementing and evaluating a youth-focused, decentralized, district-wide, and scaled-up PrEP project.

This manuscript has three aims:

1. To explore the experiences of young people involved in the youth reference group (YRG), for co-designing, implementing and evaluating a youth-focused, decentralized, district-wide, and scaled-up PrEP project.
2. To describe the staff members’ experience of YRG involvement in the FastPrEP project.
3. To provide recommendations (do’s and don’ts) for meaningfully engaging with young people when setting up and running a YRG.

## 2 Materials and methods

### 2.1 Study design and setting

Fast-PrEP was an implementation science project aiming to improve the uptake and optimal use of PrEP to adolescents and young people (15–29 years old) at high risk of HIV acquisition and with poor access to HIV prevention. The Fast-PrEP project was based in Cape Town, South Africa and provided choice for PrEP access points and/or delivery methods (facility and community-based). In addition, it introduced a choice of PrEP products beyond the current oral PrEP such as the dapivirine vaginal ring and injectable PrEP. This project aimed to evaluate whether the provision of a choice of delivery channels (with allowance for delivery channel switching) increased PrEP uptake among young people, leading to increased PrEP uptake and persistence and subsequently reduced HIV infections. In 2021, during the planning phase of this project, YRG was established to guide, co-create, monitor, and evaluate the implementation of PrEP delivery.

### 2.2 Selection of youth reference group members

The YRG consisted of 80 young people (between the ages of 16–29 years) purposively selected to represent the population of interest. Aiming for gender diversity, the YRG consisted of four sub-groups: adolescent girls and young women (AGYW), including transgender women; adolescent boys and young men; pregnant or breastfeeding women (PBFW), and young men who have sex with men (MSM). YRG members were recruited from mobile clinics, youth-based community projects, antenatal health services, and key population safe spaces. Specifically, regarding the antenatal health service, one researcher actively recruited participants who were attending the clinic by approaching them personally and inviting them to join the group. In addition, communication was sent around on existing study Whatsapp groups, explaining the new study that would be starting (FastPrEP) and inviting any interested young people who were interested to have their say in how services should be provided, to sign up for the YRG. Snowball sampling was encouraged to include a mix of PrEP-experienced and PrEP-naïve young people. The University of Cape Town Health Science Research Ethics Committee approved a waiver of parental proxy consent for those younger than 18 years (UCT HREC number 734/2020).

### 2.3 Youth engagement

**Figure 1:**
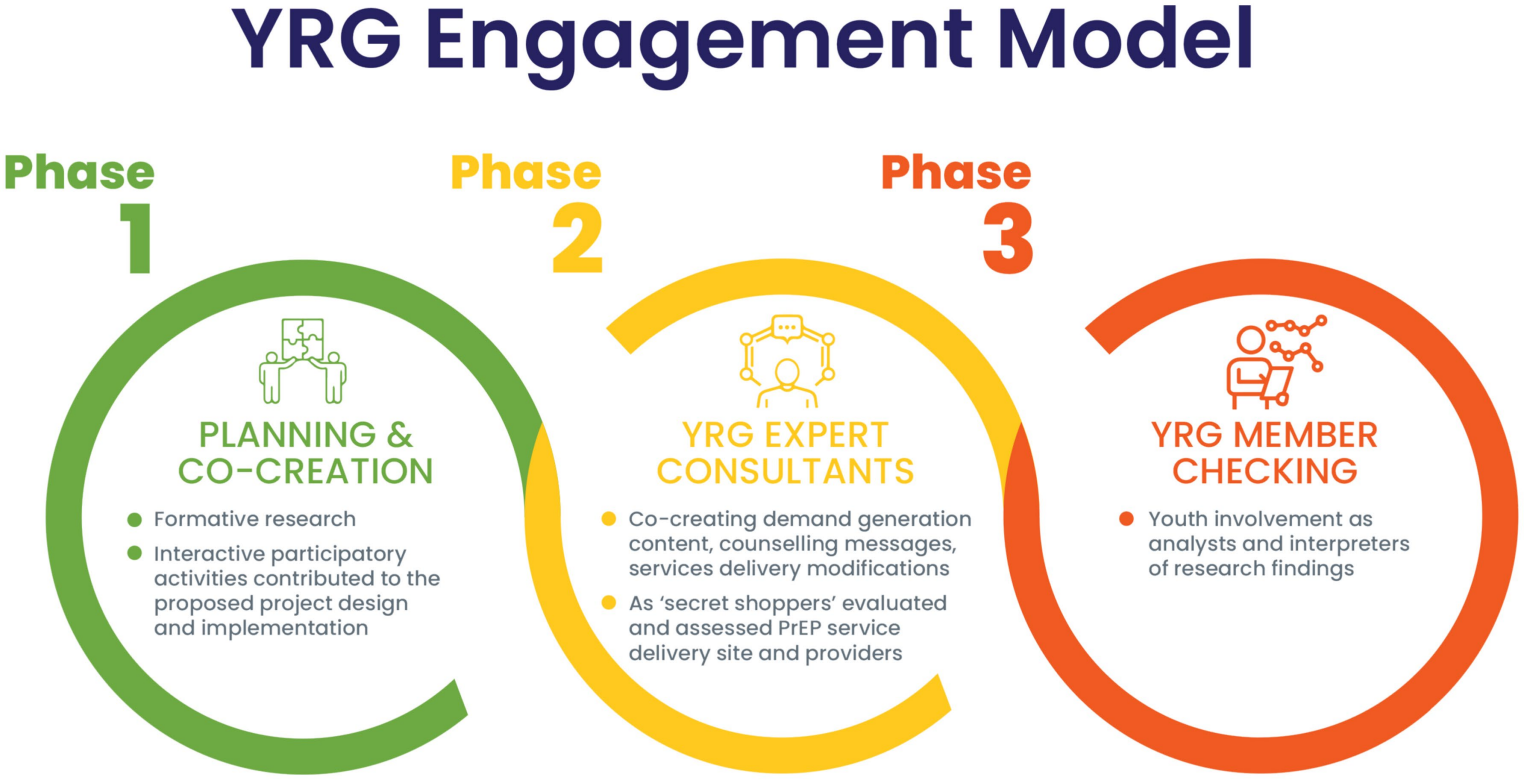
Three phrases of youth engagement.

There were three distinct phases of engagement with the YRG (Figure 1). During the first phase, *planning and co-creation*, young people attended four group discussions led by trained near-peers (an individual who is very similar to the intended beneficiary; in this case, someone close in age and from the same or similar community) that covered the topics of awareness and demand creation for PrEP, PrEP delivery platforms, and adherence support to PrEP use. By employing participatory methods such as community mapping, role plays, free listing and ranking of attributes, as well as clinic flow and PrEP-user journey diagrams, the YRG explored preferences, barriers, stigma, enablers, and influencers. Engagement with the YRG through these creative and interactive activities contributed to adaptations to the proposed project design and implementation which was launched in March 2022.

During the second phase (2022 and ongoing) the YRG represented their constituencies of young people as expert consultants by co-creating, *monitoring, and evaluating the implementation* of the Fast-PrEP project. On average, the YRG convened once a month, where they took on the role of co-designers brainstorming acceptable PrEP service delivery practices and got involved in demand creation material development. In addition, at times, members of the YRG presented unannounced to Fast-PrEP project clinics (government health facilities and community-based mobile clinics) as ‘secret shoppers’ indistinguishable by the PrEP providers from real AYP seeking services. During these visits the YRG ‘secret shoppers’ assessed PrEP service delivery site and providers regarding their skills in adolescent-friendly, patient-centered communication and provision of overall acceptable services in adherence to clinical guidelines.

The third phase of youth engagement (2022 and ongoing) focused on *member checking* including youth involvement as analysts and interpreters of research findings. This process included engagement with the YRG where the researcher solicited the YRG’s reaction to the researchers understanding of interviews or study results as an approach to accurately understand AYP’s lived experiences of PrEP use.

### 2.4 Data collection

Between March to October 2023, we conducted four focus group discussions (FGDs) (*N* = 30) and individual interviews (IDIs) with 7 project implementers to explore real-time experiences to inform best practices. FGDs were divided into four groups, one with male participants (*n* = 10) and three groups with female participants (*n* = 8, *n* = 4, *n* = 8, respectively). Of the seven project implementers, two were part of the FastPrEP clinical staff working in the mobile clinics, two were researchers and three were part of the operations team. The FGD guides included YRG experiences of consultants, best practices for researchers when engaging youth, and perceived public health impact of programs that consult with youth. Interview guides with staff included questions about their YRG engagement experiences (including benefits and challenges), perceived best practices in setting up and maintaining continuous engagement with the YRG, and recommendations on support for YRG members. The FGDs and IDIs were conducted face-to-face in isiXhosa by experienced bilingual social science interviewers who were independent of the Fast-PrEP implementation team. Each FGD and respective IDIs were audio-recorded, then simultaneously translated and transcribed.

### 2.5 Data analysis

Transcripts were analysed thematically to explore best practices and recommendations for youth engagement. Three researchers worked together to code the FGD and IDI transcripts, thereafter, grouping the codes into larger themes, until the final themes were established. A member-checking exercise then took place with young people from the YRG present. FGD interpretations and key questions were posed to the group to aid in interpreting the findings and confirming best practices for youth engagement. This feedback was then integrated with the initial analysis and further reviewed by members of the larger research team involved in the project, to produce the final manuscript.

## 3 Findings

The YRG participants’ ages ranged from 16 to 29 years old. The staff ages ranged from 31 to 50 years, with the average age being 36.6 years. The majority of the staff were female (*n* = 6) and there was 1 male. The FGDs lasted between 45 and 90 min and IDIs lasted approximately 30–60 min each. The results highlight key themes and sub-themes emerging from the discussions and IDIs, broadly covering the young people’s experiences, what it means to authentically engage with young people and certain challenges and recommendations experienced in the process.

### 3.1 Experiences of young people and staff members involved in the youth reference group

#### 3.1.1 Knowledge enrichment, empowerment and seeing ideas in practice

Involvement in the first phase of co-creation enriched young people’s knowledge of SRH, PrEP, and research, while equipping them with rights-based sexual health language. As a result, they felt that their knowledge and experience of being involved in the group equipped them to be agents of change within their communities, enabling them to approach health facilities and request their preferred SRH services.

*“We were taking wrong decisions as men. Personally after I joined those classes I made wise decision because those classes helped us. We know other things and PrEP. We know that we are supposed to use condoms so that we do not impregnate women at a certain age. I feel what R9 said is similar to what I am saying. It helped a lot.” (Respondent, Male group)*

*“It makes us to be more cautious and tell other people about what we know. It will help many people not to be infected with HIV. We help each other by attending these sessions so that we can also get knowledge on how PrEP works and pass on the information”. (Respondent 5, male group)*

Staff participants mentioned that YRG members benefited from the experience by becoming more aware of the research being conducted and gaining health literacy, which empowered them as active stakeholders.

*“…I like to think that the youth reference group also as individuals, benefit from the experience. Yeah, just by being involved, by becoming more aware of things that are being researched, by being more familiar with the research process, you know, by getting, you know, kind of passively gaining a lot of health literacy through it.” (Staff 4)*

Young people felt that their voices and needs were valued when they saw their service delivery ideas put into action or witnessed their designs in demand creation campaigns. They reported that they would have felt ‘deflated’ if their ideas were not used and heavily emphasized the need for consistent feedback and open communication.

*“I also agree that what we ask for gets done. We once had a group discussion where people were complaining about the size of the pill and all that, and even said that they’d prefer if it was an injection. I heard that a ring and an injection will be soon arriving, which means that the things people asked for will happen.” (Respondent, Female Group 1)*

*“Okay. Let’s say, for example, if you guys were consulted in those sessions, and you came up with ideas, and those aren’t implemented, how were you going to feel?” (Interviewer)*

*“I’d be deflated. What’s the point?” (Respondent, Female Group 1)*

*“Why even meet if it won’t be done.” (Respondent, Female Group 1)*

*“We’d be wasting our breath.” (Respondent, Female Group 1)*

#### 3.1.2 Authentic youth engagement: moving beyond tokenism

Young people shared that they felt they were not just consulted for tokenistic reasons, such as a required tick box with ethics committees, but for their valuable contribution to young people’s sexual health. The group appreciated that the researchers understood the importance of creating a product or service for young people by involving them from the beginning of the process. This also included involving the young people in developing ideas to reach other individuals, which included using the young people themselves.

*“I think it’s also right, because we voted, and the pamphlets are here. Which means our opinions are taken seriously in the group.” (Respondent, Female Group 1)*

*“It’s different because when you consult you’re getting the person exactly what they want. It’s better than giving them something based on an idea of what you think they want. (Respondent, Female Group 1)*

*“So, I feel like… We feel important in that way. You also respect our ideas, you see.” (Respondent, Female Group 3)*

The staff participants also emphasized the importance of making YRG members feel like active contributors throughout the study, where the young people could express their opinions freely.

*“I appreciate the honesty they have. They can freely share their opinions and ideas… I really like their transparency when it comes to us asking them questions” (Staff 7)*

Staff participants also emphasized the importance of making YRG members feel like active contributors throughout the study, creating a sense of ownership and long-term engagement.

*“It also shows that you're listening to them, you hear them, you see them, and you value their opinions. But more than anything, it encourages young people to constantly visit, you know, our mobile clinics because of our ability to implement their suggestions.” (Staff 7)*

#### 3.1.3 Staff members’ experience of YRG involvement in the FastPrEP project

Young people further reported that projects that appropriately integrate YRGs would have a wider reach and greater effectiveness in PrEP delivery to young people. Some of the clinical staff agreed, noting,

*“I can see it as beneficial in improving and engagement and accessibility for adolescent girls and young women. And also, it helps so that they can tell us what is it that they really want to see in these youth-friendly services so that they can be beneficial to their health and well-being.” (Staff 3)*

Staff participants explained that researchers benefit from involving youth as they can trial ideas and concepts before reaching participants. It also ensures that research is informed by those it is meant to benefit and acceptable to the end users.

*“I always find that they're quite clear cut in their answers and yeah just very confident about what they want to say. So I do, appreciate that in the process… And I guess in that way it is very helpful because you know, when you're planning a study and you're not sure, to be able to go to this group of people grounded in their community and then get these definite answers is very helpful.” (Staff 4)*

The staff participants found involving the YRG helpful in understanding the young people and their needs more, which helped to shape their opinion of young people in general and be aware of their actions in providing youth-friendly services. The staff participants discussed how involving the youth reference group in the development of services could lead to more tailored and effective offerings.

*“So it helps us better understand them, number one. And it also helps us to understand that they have different needs, even though they might be on the same age, but they have different needs. And it also helps us to be aware of our actions at all times.” (Staff 2)*

However, although they recognized the importance of youth consultation, some of the clinical staff noted that it is difficult to consult the youth at all times, especially when making urgent clinical decisions.

*“Obviously if it's a clinical decision that needs to be done now they won't be contacted immediately. You use your own discretion and your experience as you’re expected to as the clinician. But when it comes to the services, how part of the service should be given, if they are conducted regularly …then we'll be able to improve as the time goes on instead of us waiting [a] long [time].” (Staff 5)*

### 3.2 Potential challenges and recommendations (do’s and don’ts) when meaningfully engaging with young people while setting up and running a YRG

**Figure 2:**
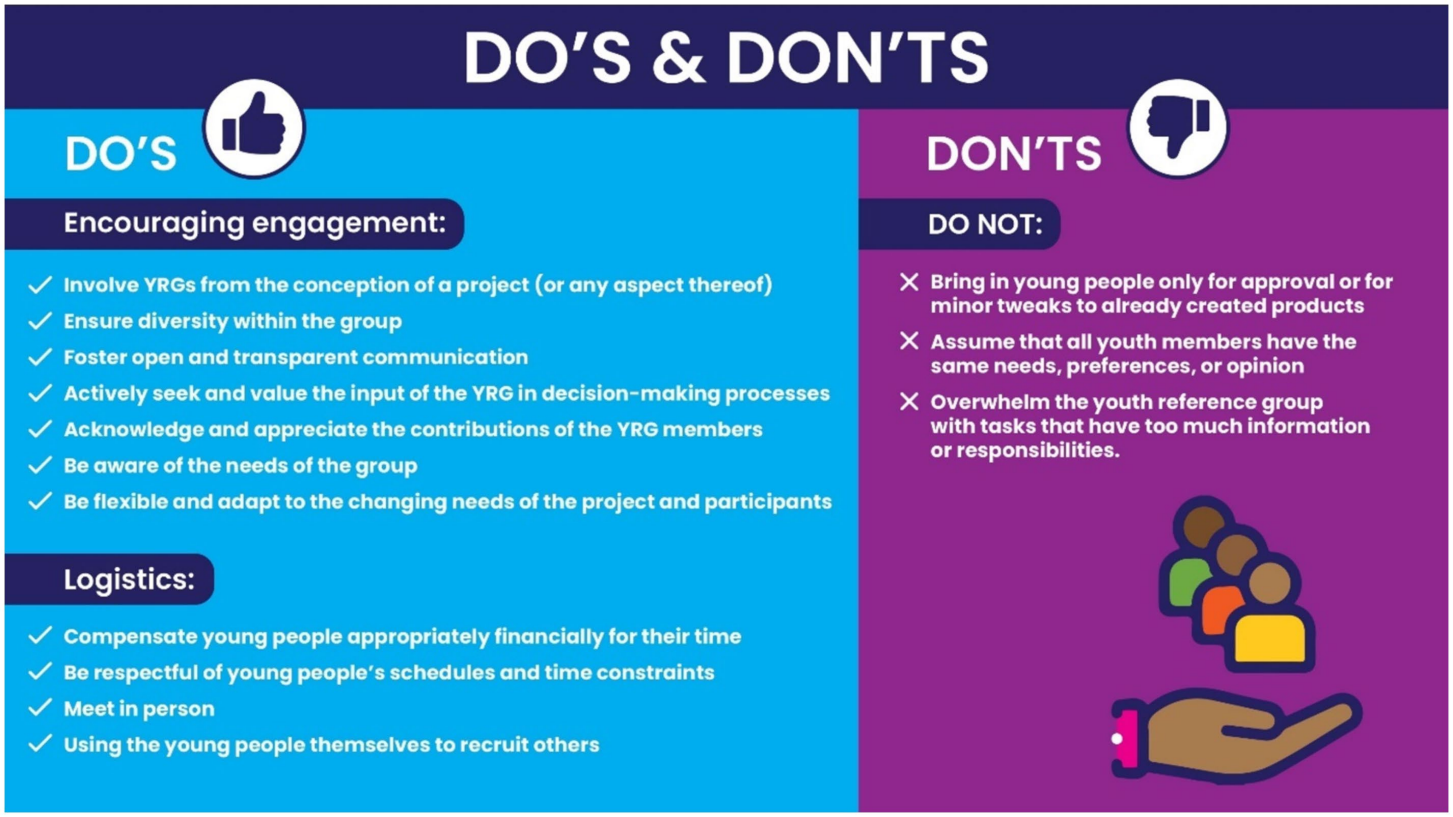
“Do’s and don’ts” for setting up and engaging with a youth reference group.

A summary of recommendations or “do’s and don’ts” for setting up and meaningfully engaging with a YRG, can be found in Figure 2.

#### 3.2.1 Challenges

Several challenges linked to logistics and lack of resources were commonly experienced when working with young people, particularly in low-income settings. Although the youth generally preferred meeting in person instead of online, they did not attend the groups as frequently as expected due to several constraints, including not having time, lack of transport money, bad weather, and lack of mobile data. Staff participants similarly mentioned challenges with poor attendance during reference group engagements, which could impact the effectiveness of the group’s contributions.

*“And, sometimes, you want to come – on the point that people aren’t coming – you want to come, but there’ll be things that come up and grab your time”. (Respondent, Female Group 2)*

Some of the research team felt that while the clinical team appreciated the young people’s input, the clinical staff did not always appreciate the youth’s feedback, as they felt personally criticized, particularly when it was regarding how the youth felt they had been treated by clinical staff. This led to the suggestion that all staff members need to have repeated training and sensitisation around the importance of youth engagement, as well as how to truly engage youth in all aspects of service provision.

*“I've picked up quite a few times that there's frustrations. For instance, the other day when we're giving feedback about some…it's how they receive things. Somehow, they feel like they've been criticised.” (Staff 6)*

*“Everyone should be able to communicate it to young people. It should not (just) be a [researcher].” (Staff 6)*

#### 3.2.2 Recommendations

Both the young people and staff members discussed a few practical tips for encouraging optimal engagement with youth. It was important to keep the young people’s schedules in mind when planning meetings, preferably having frequent but short in-person meetings on Saturdays.

*“When they are organising these sessions, they need to consider the time because we are at school and some working. (Respondent 9, male group)*

“*For me, I think, if you’re gonna start a new group, they should only come to the groups every Saturday.…Yeah, cos on Saturdays, most people are around…They need to consider time, maybe on Saturday”s. (R9, Male group)*

Groups needed to be fun, with refreshments provided and online platforms used solely for communication purposes (as opposed to online meetings using Whatsapp). Lastly, it was important to provide monetary (travel) compensation for the youth’s time, which was paid on time.

*“You should find games that people like so that they won’t be bored. Or, have a little speaker to give people some energy, and not be serious all the time…Also, brief people on what will happen…There should also be snacks, and some refreshments as we talk”. [Respondent, Female Group 1]*

*“Also, with WhatsApp, if we say we’re doing it there, people won’t respond to the messages, and some will say they don’t have data for WhatsApp, and all that. But, if we’re all here, we’ll be able to discuss”. (Respondent, Female Group 2)*

*Maybe the person will have transport challenges coming here because of money. It will be better if they provide transport or give them money for transport so that they can come. (R5, male group).*

Some staff mentioned that Youth-to-Youth Engagement emphasised the value of using younger individuals to engage with participants, as they are more relatable and have the language and skills to connect with youth effectively.

*“I think they can break the stigma, yes, because they talk on behalf of the youth…. They will help the other youth to have accurate information about PrEP and other health related discussions, help eliminate fears and also help their peers to gain kind of support and encouragement.” (Staff 3)*

Some staff expressed concerns about the depth of discussions within the youth reference group and how some dominant voices tended to dominate the conversation. This could hinder the participation of less confident members with valuable, nuanced perspectives.

*“The conversation sometimes just doesn't grow deep enough for me to feel fully satisfied that we've really unpacked the issue. And that's part of the reason I started trying to send these 2 pages before to give people time to think about it.” (Staff 4)*

Suggestions for combatting this included allowing young people to lead discussions and presentations about different topics and preparing and providing specific support and training where required.

The staff had several recommendations for training, information and tools that the YRG would benefit from, to better equip them for being part of the YRG and to assist them in their personal circumstances. Practically, they felt that the young people would benefit from training on the basics of PrEP and HIV and the research process in general.

*“I think apart from giving them the online training about Prep, we should also be training them about other HIV related courses that are for non-clinicians so that they are well equipped with everything that that pertains to HIV prevention.” (Staff 3)*

*“I think the obvious one is… research training workshops or anything that will upskill their knowledge about research, their knowledge about the content that we usually present on.” (Staff 4)*

They also suggested focusing on developing certain ‘soft skills’ such as building self-confidence and communication skills required for certain jobs such as peer navigators or HIV counselors. Public speaking, problem-solving, and decision-making skills.

*“… and if we could have programs that are going to help them upskill themselves, [if] they're going to be navigators in the future or whether they're going to be councillors in the future.” (Staff 2)*

*“In terms of in terms of facilitation for instance, I don't know if there [are] facilitation programs that we can get them into. I don't know if maybe we should have sessions like for instance, self-esteem sessions with them that could also help boost their self-esteem or self-confidence.” (Staff 6)*

#### 3.2.3 Diversity and inclusion

Among the young people, there was a mix of views when it came to inclusion and diversity of the groups. One female group member suggested that she would like to have a mix of male and female group members, whereas the male group were leaning more toward being in group discussions with peers only.

*“If its men, we can freely talk because we understand each other as men”. (Respondent 2, male group)*

Staff participants raised the issue of the underrepresentation of certain groups in the youth reference groups. They stressed the importance of having diverse perspectives to ensure inclusivity and comprehensive decision-making. The YRG serves as a means to improve accessibility for all young people; however, staff raised concerns about representation and ensuring that the voices of all youth are heard.

*“It's making the team diverse and inclusive in that it's only we're only seeing heterosexual females and males, but we don't have many from the LGBTQI community. So young people of that community, for us to be able to know for sure that our services are really speaking to them.” (Staff 7)*

## 4 Discussion

### 4.1 Involving youth in the research process

Involving youth in the research process and shaping of clinical services has numerous benefits for both young people and service providers. Other youth-led initiatives have found that young people benefit by being peer educators, allowing them to have more knowledge about the subject and developing a sense of agency and personal efficacy from knowing their ideas are listened to and implemented (13). Similarly, our findings suggest that while the primary aim of the YRG was to provide feedback on the youth-led services, one of the main by-products was the increased PrEP knowledge and empowering sense of agency in the young people. This is particularly relevant as engaging and empowering people and communities is one of the five implementable strategies that the WHO advises, to achieve their framework on integrated people-centred health services (5).

There is a common misconception that including lay people in research planning, particularly young people, can slow down the research process, as things may need to stop and start according to when the input is required. However, if implemented correctly (i.e., properly engaging with young people from the beginning of the process), the process can be simple and effective, often resulting in researchers being able to plan and implement projects tailored to the specific community in which they are working. This was similarly observed by the researchers in the study, who found it helpful to trial and test ideas before introducing them to the study participants. Furthermore, youth-led research has been found to have direct benefits for the research process, including higher quality of the research itself, with data collection tools having user-friendly language and more insight when interpreting findings (13). Our findings support this, with researchers finding benefits in including youth in the process from the beginning, making sure that research was relevant and appropriate for the intended end users.

### 4.2 Involving youth when planning and implementing PrEP service provision

For services to be accessible to different people groups, they need to be approachable, acceptable, available, affordable and appropriate (6). Although all primary (including sexual and reproductive) health services in South Africa are provided free of charge to users, there are often direct (e.g., transport costs) and indirect (e.g., taking time off work/school) costs that can impact the affordability of the service (7). Engaging with young people can provide meaningful insight into knowing the financial barriers as well as potential solutions to encourage greater access. Some ideas that have been implemented in the greater FastPrEP study include the use of alternative delivery methods (such as courier services) and providing mobile services close to young people’s homes or schools. These adapted methods of service provision encourage greater availability of PrEP and have been found to be effective with other chronic medications in South Africa (14). The concepts of ‘approachability’, ‘acceptability’ and ‘appropriateness’ are particularly important when it comes to youth-friendly services (15). Clinic environments need to feel safe and welcoming, and staff should be respectful, use polite gestures and practice open communication (15). Engaging with young people provided insight for the staff to provide tailored and effective services, making them more acceptable and appropriate to the end users. Opportunities for consistent training, feedback, engagement with young people and sensitisation to the needs of young people need to be provided in all youth-friendly organizations to encourage health workers to see their end users as equal partners in service provision (7).

### 4.3 Recommendations

While there was some conflict in opinions among the young people, as to whether the groups should be more inclusive or not, it is generally recommended that all young people’s opinions and feedback are unique, and a diversity of socio-demographics should be considered when recruiting group members (15). This is particularly important for smaller under-represented groups, such as members of the LGBTQI+ community. However, careful considerations do need to be made around how welcoming certain individuals and groups will be if a diverse group is mixed together. Furthermore, as it is difficult to integrate everyone’s feedback, it can be helpful to only include group members with similar demographics to the end user target population (15).

Researchers and clinical service providers both benefit from involving end users, from the conception right through to the evaluation of a project or service. Many AGYW in lower- and middle-income countries (LMICs) live in a context where gender norms, power dynamics and other social determinants of health impact their decision-making power, education and economic security, particularly when it comes to access to SRH services (16). It is, therefore, imperative that engagement with young people is prioritized, where their suggestions and ideas are taken seriously and clearly put into practice. However, youth engagement comes with its own challenges, including managing group dynamics and facilitating meaningful engagement. These challenges can be mitigated by regularly bringing in new members of the group to bring fresh and diverse opinions and making use of trained facilitators to encourage rich and meaningful discussions and feedback (17).

### 4.4 Study limitations and conclusion

There are a number of limitations synonymous with conducting qualitative research, and specifically participatory research. Although the group members were similar in age, ethnicity and socioeconomic status, no group is ever homogeneous and therefore some of the research findings may not be generalisable to other contexts. However, many of our findings are in line with literature from other settings, and most of the themes and recommendations could be applied in most youth specific contexts. When conducting research with certain groups, particularly with young people, there needs to be an element of trust between the group members and group facilitators to elicit optimal engagement. As a result, those conducting the focus groups and interviews were well known to the respective participants, which may have created the potential for bias. This was minimized using researcher triangulation to analyse results and member checking with members of the YRG during the different phases of the research process. The researchers acknowledge that the sample of staff members was small, and while adequate data saturation was reached, including more clinical providers could have added more nuanced insight.

Including young people in the set up and implementation of a research project or health service can be empowering for young people themselves and beneficial in providing services that are accessible and acceptable for end users. Best practices include involving the YRG in every aspect of the project design and implementation. However, this process can be perceived as an obstacle to rapid implementation, especially by the clinical team (nurses), who can be unaccustomed to relying on patient or client feedback and direction for their service provision. Research and clinical staff need ongoing training and sensitisation on the importance and value of youth engagement. Projects that appropriately integrate YRGs will have a wider reach and greater effectiveness in PrEP delivery to young people.

## Data Availability

The raw data supporting the conclusions of this article will be made available by the authors, without undue reservation.
